# A Mislocated Intrauterine Device Migrating to the Urinary Bladder: An Uncommon Complication Leading to Stone Formation

**DOI:** 10.1155/2020/2091915

**Published:** 2020-04-07

**Authors:** Mohamed Ali Nouioui, Tarek Taktak, Seif Mokadem, Houssem Mediouni, Ramzi Khiari, Samir Ghozzi

**Affiliations:** Department of Urology, Military Hospital of First Instruction of Tunis, University of Tunis El Manar, Tunisia

## Abstract

Intrauterine devices are a popular form of reversible contraception among women. Its administration can lead to some uncommon but serious complications such as perforation leading to its migration into adjacent organs. Like any foreign body, the presence of an IUD in the bladder can result in stone formation due to its lithogenic potential. We report a case of an IUD migrating from its normal position in the uterine cavity into the urinary bladder causing chronic low urinary tract symptoms in a 43-year-old female patient. The device was securely removed without complications using grasping forceps under cystoscopy, and no parietal defect was detected. A mislocated IUD is a rare complication that should be considered in female patients presenting with chronic urinary symptoms.

## 1. Introduction

Intrauterine devices (IUD) are a frequently used reversible form of contraception.

As any medical device, its administration can lead to complications such as perforation and extrauterine migration to adjacent organs.

The most common sites for IUD migration are the omentum, rectum, sigmoid colon, peritoneum, and bladder [1].

Here, we report a case of an IUD migrating from the uterus cavity into the urinary bladder.

## 2. Case Presentation

A 43-year-old female was referred to our outpatient department for a one-year history of chronic pelvic pain with lower urinary tract symptoms such as dysuria, urgency, and pollakiuria.

Her medical history was unremarkable other than the insertion of a Copper-T IUD four years ago by her gynaecologist and with no regular follow-up afterwards.

Physical pelvic examination was normal.

Urinalysis was indicative of leucocyturia, but urine culture was negative.

**Figure 1:**
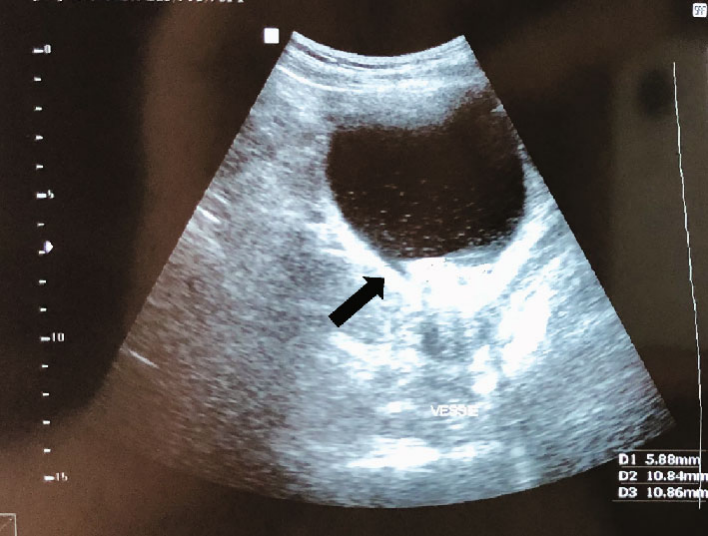
Pelvic US revealing an echogenic intravesical foreign object (arrow) initially suggestive of urinary bladder calculus.

An abdominal ultrasonography revealed an echogenic intravesical lesion suggestive of urinary bladder calculus (Figure 1).

**Figure 2:**
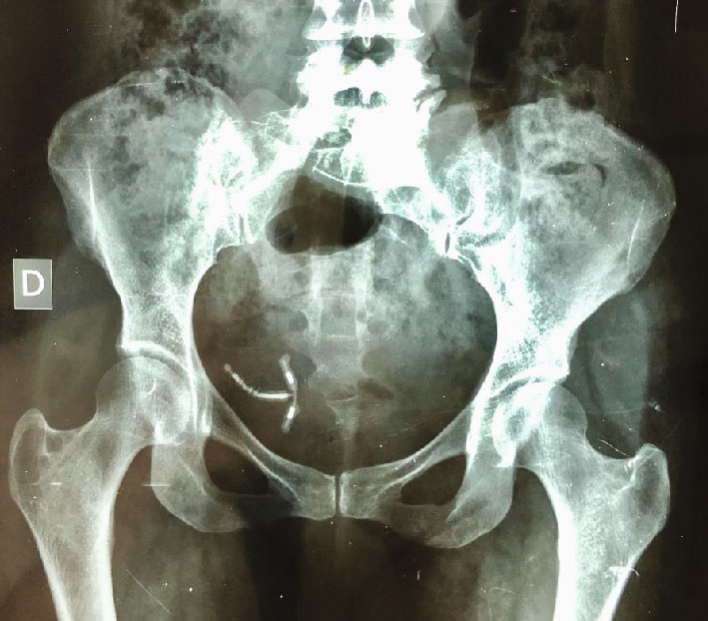
A mislocated IUD in the lower left quadrant of the abdomen showed on the plain abdominal X-ray.

Plain abdominal radiography did not show any stone but demonstrated the IUD in the lower left quadrant of the abdomen (Figure 2).

A cystoscopy was done after written informed consent of the patient. It revealed a calcified T-shaped foreign object identified as the IUD, embedded into the muscular wall of the bladder.

No stones were found.

The IUD was extracted easily with gentle traction using endoscopic forceps.

After the removal of the foreign object, we did not find any fistula tract between the posterior wall of the bladder and the uterus.

One month following the procedure, the patient was reassessed and reported a significant improvement in lower urinary symptoms including no evidence of infections in the urine culture or persistence of fistula at the flexible cystoscopy.

## 3. Discussion

Intrauterine device is a regular form of mechanical contraception widely used. More than 150 million women use IUDs, mainly in emerging countries [2].

Its administration can be accompanied by several complications.

Perforation is one the most serious but rare complication secondary to the insertion of IUD, eventually leading to its migration from its normal position in the fundus either into the abdominal cavity or into other organs adjacent to the uterus.

The incidence of perforation is estimated as being between 1.9 and 3.6 per 1000 insertions [3].

The true incidence of perforation is most likely higher because of the frequently asymptomatic nature of the perforation, with over 30% of perforations recognized only when pregnancy [4].

The urinary bladder is one of the organs where the mislocated IUD can be embedded.

Table 1 shows five similar cases with different therapeutic approaches reported in literature.

Bladder stones (BS) are uncommon in women. Approximately, 5% of bladder stones occur in female patients [5].

Therefore, foreign bodies should be considered when assessing the presence of BS.

These foreign bodies such as IUD in this case, with their lithogenic potential, act as a nidus for stone formation in the urinary bladder [6].

Although perforation of the uterus by IUD is often a silent phenomenon, erosion of the bladder wall is usually symptomatic [7].

In this case, the patient presented with irritative low urinary tract symptoms, such as frequency and urgency, frequently associated with haematuria.

Recurrent urinary tract infections, chronic pelvic pain syndrome, and sexual complaints such as dyspareunia can be part of the clinical manifestations.

Typically, at pelvic examination, the retrieval string of the IUD should protrude approximately 2-3 cm through the external cervical os.

A missing string is a common and strong indication of displacement, uterine perforation, or expulsion.

Various imaging modalities can be used in the evaluation of IUDs and in establishing the etiological diagnosis of low urinary tract symptoms in female patients.

Abdominal ultrasonography (US) can easily help determine whether the IUD is correctly positioned.

IUD displacement and myometrial perforation can be fully investigated by performing US alone [8].

However, abdominal radiography can be helpful in demonstrating an extrauterine IUD.

In some cases, the IUD can be misdiagnosed for a bladder stone due to the calcium tone opacity acquired when intravesical.

Thus, diagnostic cystoscopy allows a direct visualisation of the bladder, allowing to identify the foreign body, its extraction using a mechanical forceps if possible, and the search for any vesicouterine fistula.

However, the management of migrated IUDs is controversial.

According to the World Health Organization (WHO) recommendation, any translocated IUD following uterine perforation within the abdomen should be removed whether symptomatic or asymptomatic irrespective of location [9].

An IUD that migrated to the bladder eventually leads to stone formation, making its removal necessary.

Three approaches to remove the device include the use of open cystolithotomy, transurethral grasping forceps, or minimally invasive laparoscopy [10].

Open and laparoscopic surgery should be considered for the removal of IUD with partial penetration due to the possibility of a vesicouterine fistula that needs repair [7].

In case of endoscopic approach, both the cystoscope and the transurethral nephroscope can be used.

Ballistic or laser lithotripsy should be used for the initial fragmentation of the calculi that formed around the IUD to facilitate its extraction.

In this case, cystoscopy was sufficient for the management of this mislocated IUD.

Although delicate surgical procedures are available nowadays, traditional noninvasive options such as herbal formulas have been reported for patients living in developing areas with known or unidentified mechanisms of actions, but an evidence based practice should be kept in mind [11].

## 4. Conclusion

IUD migrating to the urinary bladder is an uncommon complication that should be considered in female patients complaining of chronic low urinary tract symptoms.

Endoscopic approach of a mislocated IUD is considered a safe and effective minimally invasive approach.

## Figures and Tables

**Table 1:** Review of intravesical migration of IUD cases reported in English language literature.

Author and year	Age	Time of IUD insertion	Clinical presentation	Urinalysis	Imaging performed	Radiological findings	Calculus formation	Peri-op findings	Treatment	Outcome and Follow-up
Niu et al., 2018	57	26 years ago	Chronic supra-pubic pain	Unremarkable	Plain abdominal X-ray US	Hyperechoic foreign body penetrating through the anterior uterine wall into the posterior supratrigonal bladder wall	On the tip of the penetrating IUD	Shifted IUD horizontal limb protruding through the anterior uterine wall into the bladder. Needle-like calculus on the IUD.	Endoscopic approach: Laser fragmentation of the calculus encapsulating the IUD. Removal of the IUD with forceps through the nephroscope. Placed in the uterine cavity, 2 weeks indwelling Foley catheter.	No subsequent evidence of residual perforation or fistula
Shin et al., 2011 [12]	38	11 years ago	Recurrent UTI dysuria, dyspareunia chronic pelvic pain	Leucocyturia	Plain abdominal Radiograph Pelvic CT	Stone opacity around the IUD 1.6 × 1.9 cm stone around the IUD located in the bladder	Yes	IUD deeply embedded into the muscular bladder wall. No vesico-uterin fistula	Laparoscopic approach: Removal of the IUD and the bladder stone via laparoscopic cystolithotomy	No leakage on cystography.
Yahsi et al., 2015 [13]	37	6 years ago	Supra-pubic pain Polyuria, urgency	Leucocyturia	Plain abdominal x-ray Pelvic US, CT	1,5 × 2 cm bladder stone on the IUD whose 1 cm tipn outside of the baldder.	Yes	Intravesical encrusted IUD entering the bladder lumen posteriorly.	Endoscopic approach: IUD stuck to the bladder. Removal with cystoscopic forceps failed. Laparoscopic approach.	Discharged after normal post-op cystography.
De Silva et al., 2017 [14]	48	15 years ago	Recurrent UTI Dysuria, hematuria	Proteus growth	Plain abdominal X-ray US	Intra-vesical large stone with three limbs and an imprint of a typical IUCD in the middle of the stone.	Yes	6 × 5 cm bladder stone with three limbs shaped to cover the IUCD found inside.	Open cystolithotomy.	Uncomplicated post-op period, asymptomatic afterwards, no further..
Gharbi M. et al., 2019 [15]	62	9 years ago	Intermittent pelvic pain	Pyuria, hematuria	Plain abdominal X-ray US	15-mm calcified pelvic mass overlying the copper-T echogenic intravesical lesion with distal acoustic shadow.	Yes	Large calculus at the end of the IUD wire penetrating the posterior wall of the bladder.	Endoscopic approach: Ballistic lithotripsy of the stone removal of the IUD after calculi fragmentation.	Uncomplicated post period.
